# Anterior ischemic optic neuropathy in a patient with optic disc Drusen while on FOLFOX Chemotherapy for colon cancer: the value of Occam’s Razor and Hickam’s dictum

**Published:** 2019

**Authors:** Gehad Ayman Elnahry

**Affiliations:** * Department of Ophthalmology, Faculty of Medicine, Cairo University, Cairo, Egypt

**Keywords:** Anterior ischemic optic neuropathy, FOLFOX chemotherapy, Hickam’s dictum, optic disc drusen

## Abstract

A 53-year-old male developed acute diminution of vision in his right eye while on FOLFOX chemotherapy for stage C colon cancer. Examination revealed bilateral optic nerve head swelling with flame shaped hemorrhages over the right optic disc and anomalous left retinal vasculature. Computed tomography scan of the brain and orbit revealed no cerebral pathology, however bilateral optic disc drusen (ODD) was suspected. B scan ultrasonography confirmed the presence of bilateral ODD. Fluorescein angiography showed early hypofluorescence of the right optic disc with bilateral late disc staining and a diagnosis of right anterior ischemic optic neuropathy (AION) with bilateral ODD was made. A literature review was performed and possible mechanisms for the development of AION in this case were discussed.

## Introduction

Anterior ischemic optic neuropathy (AION) is a serious vision threatening condition that may affect any age group but is more common in middle aged and elderly patients [ **1**]. It may be arteritic (AAION) or non-arteritic (NAION) and occurs due to ischemia of the optic nerve head, which normally receives its blood supply through the posterior ciliary arteries [ **2**]. While AAION mainly occurs with giant cell arteritis, NAION is multifactorial, with several reported predisposing and precipitating factors [ **1**]. We report a case of NAION that occurred in a patient with optic disc drusen (ODD) while on FOLFOX chemotherapy (5-fluorouracil, oxaliplatin, and folinic acid) for colon cancer treatment.

## Case report

A 53-year-old male patient with a history of stage C colon cancer complained of painless diminution of vision in his right eye of acute onset and stationary course for 3 days. He underwent a hemicolectomy for his colon cancer 5 months before and had been receiving chemotherapy in the form of 5-fluorouracil, oxaliplatin, and folinic acid every 2 weeks for 4 months. He had a past medical history of hypertension, which was controlled by medication. He had no previous history of ocular problems.

**Fig. 1:**
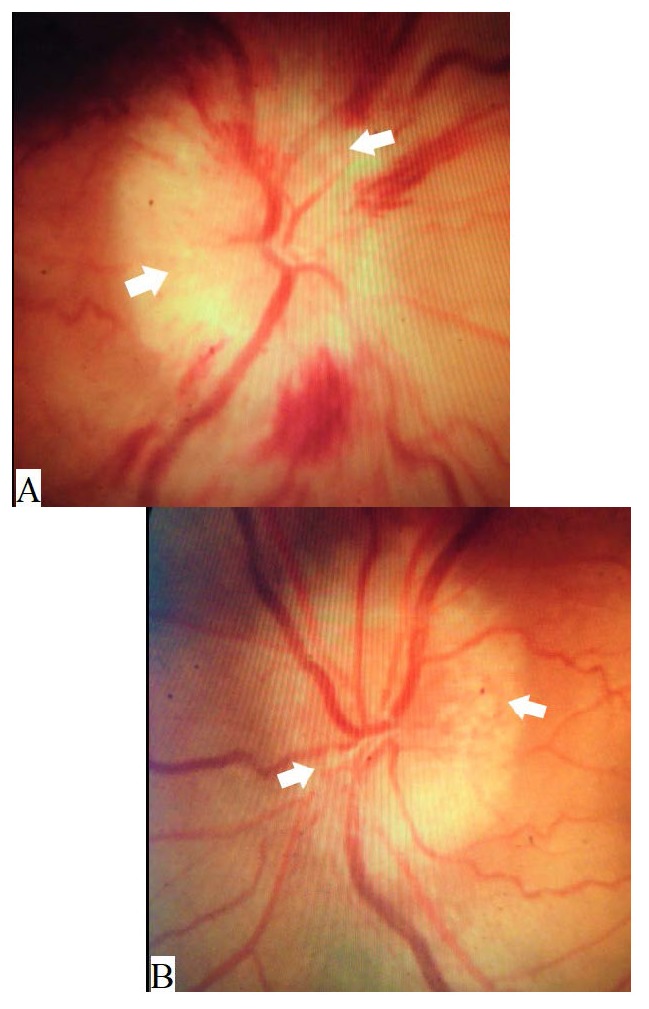
Color photography of the optic discs showing swelling of both optic discs with blurred margins more temporal than nasal and multiple peculiar nodules (white arrows) suggestive of optic disc drusen, with multiple flame shaped hemorrhages over the right disc (A) and an anomalous left retinal vasculature (B)esence of a large “cotton-ball” colony in the patient’s right eye

Examination revealed a corrected distance visual acuity of 20/ 400 in the right eye and 20/ 80 in the left. Color vision was 2/ 10 in the right eye and 10/ 10 in the left using Ishihara plates. Examination of the pupil revealed a right relative afferent pupillary defect. Intraocular pressure was 16 mmHg in both eyes. Extraocular motility was full. Anterior segment examination revealed nuclear cataract in both eyes. Fundus examination showed bilateral optic disc elevation with multiple superficial nodular swellings over both optic discs. There were also multiple flame-shaped hemorrhages over the right optic nerve head with an anomalous left retinal vasculature ( **Fig. 1**).

**Fig. 2:**
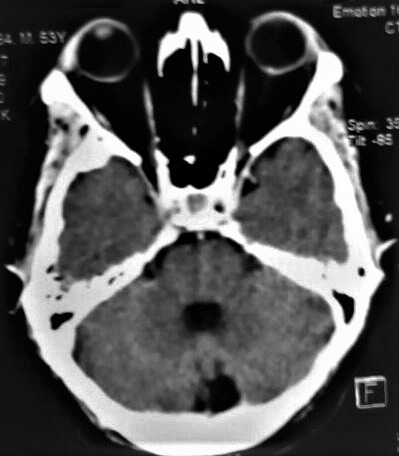
Computed tomography scan of the brain and orbit showing hyperdense lesions in both optic nerve heads larger on the left side

A computed tomography scan of the brain and orbit was performed which revealed no cerebral pathology but suggested the presence of bilateral ODD more on the left side ( **Fig. 2**).

**Fig. 3:**
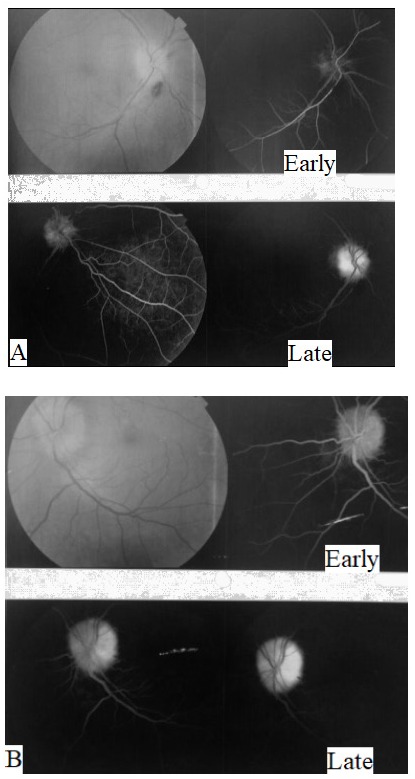
Fundus photography and fluorescein angiography of both eyes. A. The right optic disc was swollen with flame shaped hemorrhages, and fluorescein angiography showed early disc hypofluorescence indicating hypoperfusion with late staining and mild leakage. B. The left optic disc was swollen with anomalous retinal vasculature, and fluorescein angiography showed early disc hyperfluorescence with late staining and no leakage side

Right NAION on top of bilateral ODD was suspected and fundus fluorescein angiography was subsequently performed to confirm the diagnosis ( **Fig. 3**).

**Fig. 4:**
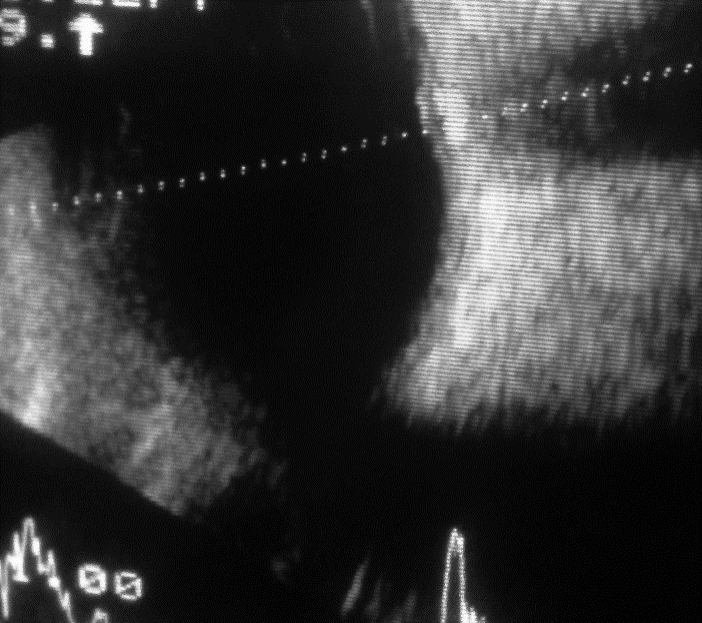
4 B-scan ultrasonography of both eyes showed optic disc elevation with the presence of buried drusen

B scan ultrasonography was also performed to confirm the presence of ODD and revealed bilateral buried drusen ( **Fig. 4**). Visual field testing revealed an altitudinal field defect in the right eye involving the inferior hemifield. A final diagnosis of bilateral ODD with right NAION was made and diminution of vision in the left eye was attributed to the presence of nuclear cataract and ODD. Follow-up of the patient a few months later revealed right optic disc pallor with no improvement in visual acuity.

## Discussion

ODD are calcified deposits that cause elevation of the optic nerve head and mimic optic disc edema (ODE) when buried. Diagnosis of ODE on top of ODD can be even more challenging, with fluorescein angiography playing an important role in the diagnosis. There is typically leakage of fluorescein from the optic disc in cases of ODE, while only staining is present in ODD. The presence of clinical evidence of ODE, as flame-shaped hemorrhages in our case, may also help in the diagnosis [ **3**]. ODD have been reported to predispose to NAION due to the associated anatomical abnormalities and the mechanical compression of the surrounding vessels by the deposits [ **1**, **4**, **5**]. Periods of hemodynamic instability, such as during pregnancy, may also serve as a contributing factor to the development of NAION in patients with ODD [ **6**].

FOLFOX chemotherapy is a combination of 5-fluorouracil (5-FU), oxaliplatin and folinic acid that is commonly used in the treatment of colon cancer. A previous case report suggested that FOLFOX chemotherapy may cause NAION through the vasospastic effect of 5-FU, but the patient had no other detectable risk factors for developing NAION. No photographic or angiographic evidence of NAION was provided in that report [ **7**]. Similar to anemia or hemodynamic instability during pregnancy, it is plausible to suspect that a vasospastic effect caused by 5-FU could also lead to NAION in optic nerves with already compromised blood flow due to ODD [ **6**]. 5-FU is usually given as an infusion over a few days and is available as several generic brands. The author have observed an increased incidence of visual complaints with certain generic brands of 5-FU but not others during infusion in one patient (Unpublished data). The patient complained of multiple attacks of transient vision loss in one eye during the infusion period, which may have been due to transient optic nerve or retinal ischemia due to presumed vasospastic attacks caused by 5-FU. There were no structural optic nerve abnormalities in that case. Another possible mechanism for NAION occurring during FOLFOX chemotherapy includes hypotensive episodes that occur due to the administration of vasodilators as nitrates for prophylaxis against coronary vasospasm caused by 5-FU [ **8**]. Hypotensive episodes, a known precipitating factor for NAION, may lead to a fall in blood pressure and blood flow to the optic nerve head leading to ischemia [ **1**]. Another report of visual complaints with FOLFOX chemotherapy suggested oxaliplatin as a possible cause of ocular toxicity, unlike our case however, the ocular findings were not suggestive of NAION [ **9**].

According to Hickam’s dictum, “a patient can have as many diagnoses as he darn well pleases”, meaning that more than one disease may be responsible for a patient’s presentation. On the other hand, in the 14th century, William of Occam suggested that “plurality must not be posited without necessity”, which implies that “among competing hypotheses, favor the simplest”, this became known as “Occam’s razor” [ **10**]. These concepts are important to know and were beneficial in helping to reach the diagnosis in our case, especially Hickam’s dictum, as the patient did indeed turn out to have multiple diagnoses at the same time, including colon cancer, optic disc drusen, and NAION. Occam’s razor, however, was helpful in realizing the possible association between NAION, optic disc drusen, and FOLFOX chemotherapy.

This report suggests that further studies are required to determine the risk and mechanism of developing NAION in patients on FOLFOX chemotherapy, especially in those already predisposed to such event by other conditions such as ODD. Detection of such an association may alter the choice of chemotherapy or allow a closer ophthalmologic follow up for certain patients who might be at a higher risk of developing NAION while on FOLFOX chemotherapy such as hypertensive patients, patients with small optic discs, or patients with ODD [ **1**, **1**, **2**, **5**]. If such an association is established, therapies aiming at reversing the vasospastic effects of 5-fluorouracil, or measures employed to raise the blood pressure during hypotensive episodes, may prove useful.

**Ethical approval**

This report has been approved by Cairo University research ethics committee and adhered to the tenets of the Declaration of Helsinki.

**Financial support**

None.

**Conflicts of Interest and Source of Funding**

None.

**Acknowledgement**

None.
